# A Novel Beamforming Algorithm for GNSS Receivers with Dual-Polarized Sensitive Arrays in the Joint Space–Time-Polarization Domain

**DOI:** 10.3390/s18124506

**Published:** 2018-12-19

**Authors:** Haiyang Wang, Zhicheng Yao, Jian Yang, Zhiliang Fan

**Affiliations:** 1 Department of Missile Engineering, Rocket Force University of Engineering, Xi’an 710025, China; haiyang_wxdh@163.com (H.W.); why_gnss@outlook.com (Z.Y.); epgc_gnss@163.com (Z.F.); 2 Department of Electronic Engineering, Xidian University, Xi’an 710071, China

**Keywords:** GNSS receiver, array antenna, dual-polarized sensitive array (DPSA), interference mitigation, space–time-polarization adaptive processing (STPAP), beamforming

## Abstract

Dual-polarized sensitive arrays (DPSAs) with the space–time-polarization adaptive processing (STPAP) technique, which employs the polarization domain as well as the space domain and time domain to filter out interferences, can cancel a larger number of wideband interferences for GNSS receivers. However, the traditional STPAP beamforming algorithm, which requires a separate adaptive filter for each GNSS satellite, will make the process computationally intensive as there are multiple GNSS satellites in the field of view (FOV). In order to overcome the shortcoming, a novel STPAP beamforming algorithm based on the minimum variance distortionless response (MVDR) criterion is proposed. Compared with the traditional STPAP beamforming algorithm, the proposed STPAP beamforming algorithm can process multiple GNSS satellites at once using only one adaptive filter, which will greatly reduce the computational complexity. Moreover, the proposed algorithm will not lead to a sharp deterioration in the output carrier-to-noise density ratio (C/N_0_) performance if the number of GNSS satellites processed in the same adaptive filter is proper. Furthermore, to calculate weight vector iteratively, an adaptive algorithm based on the constrained least mean square (CLMS) method is derived for the proposed STPAP beamforming algorithm. Simulation results validate that the proposed algorithm is effective in mitigating interferences for GNSS receivers in the joint space–time-polarization domain and meanwhile has lower computational complexity when maintaining the output C/N_0_ performance close to that of the traditional STPAP algorithm.

## 1. Introduction

GNSS has been widely applied in both military and civil fields because it can provide all-time, all-weather, and high accuracy position, navigation, and timing service to global users. Although the power of the GNSS signal is 20 dB lower than the ambient noise floor, GNSS receivers can withstand a certain level of interference due to the direct sequence spread spectrum (DSSS) technique used in GNSS [1,2,3,4,5,6,7,8,9]. However, GNSS receivers will be interfered when interferences are strong enough. Antenna array processing technique provides an effective method to cancel interferences [10,11,12]. The concept is to apply weights on the signals received by different array elements to form nulls towards the arriving directions of incoming interferences while steering the array response towards the desired GNSS signals. 

Antenna array with the space adaptive processing (SAP) technique performs well in cancelling interferences and preserving GNSS signals [13,14,15,16], while it also has disadvantages. At first, the SAP can only filter out M−1 interferences with an array antenna consisting of M elements. Nevertheless, in the multipath interference environment, the number of interferences increases dramatically and thus the performance of the SAP will be greatly deteriorated. Moreover, GNSS signals may be weakened as well when their directions of arrival (DOAs) are close to those of interferences. To overcome these shortcomings of the SAP, the space–time adaptive processing (STAP) technique was proposed in [17,18,19,20,21]. In the STAP, the temporal filter is adopted by placing a finite impulse response (FIR) filter behind each array element. Consequently, the STAP can cancel MK−1 narrowband interferences or M−1 wideband interferences if array antenna with M elements that followed by K order FIR filter is utilized. In addition, interferences incoming from directions close to those of the desired GNSS signals can also be rejected without mitigating GNSS signals if their frequencies are not identical to those of the desired GNSS signals. 

Although the STAP has combined the space domain and time domain to cancel interferences, the polarization domain is another field that can be utilized to distinguish and suppress interferences. In [22,23,24,25,26,27], the space-polarization adaptive processing (SPAP) technique was introduced to filter out interferences in the joint space-polarization domain while the time domain is ignored. Furthermore, the STPAP technique was proposed in [28,29], which can mitigate interferences for GNSS receivers in the joint space–time-polarization domain. Using DPSA with M elements that followed by K order FIR filter, the STPAP can cancel up to 2MK−1 narrowband interferences or 2M−1 wideband interferences. It indicates that the STPAP can cancel more interferences than the SAP, STAP, and SPAP with the same size array antenna, which is of great significance for platforms that can only vacate small place for equipping GNSS array antennas. 

To implement the STPAP technique, several criterions to derive weight vector for DPSA have been proposed in [29,30], which can be summarized as follows: (a) power minimization (PM) criterion. The PM criterion is easily put into practice as it just keeps the reference channel undistorted and does not need any prior knowledge, but its C/N_0_ performance is worse than the other three criterions. (b) Minimum mean square error (MMSE) criterion. The MMSE criterion performs better than the PM criterion, while it makes the process computationally intensive since each GNSS satellite in the field of view (FOV) requires a separate adaptive filter. (c) Minimum mean square error averaged over hemisphere (MMSE-AH) criterion. Compared with the MMSE criterion, the MMSE-AH criterion achieves less computational complexity but withstands C/N_0_ performance degradation. Meanwhile, the MMSE-AH criterion gives more complicated computation and only marginally better C/N_0_ performance than the PM criterion. However, the MMSE criterion and MMSE-AH criterion are difficult to be carried out in practice as the desired GNSS signal is supposed to be accurately known, due to which we will not take these two MMSE criterions into consideration in the following work. (d) Minimum variance distortionless response constraining single satellite (MVDR-CSS) criterion. In this method, the constraint vector is set to constrain only one GNSS satellite at once, in which beam forms towards the desired GNSS satellite and thus the C/N_0_ performance is greatly improved. Nonetheless, the MVDR-CSS criterion also cannot avoid complicated calculations when there are multiple GNSS satellites in the FOV because each GNSS satellite needs a separate adaptive filter. Besides, a very interesting and valuable study is introduced in [31], in which a beamforming algorithm for multiple simultaneous desired signals is proposed. In this work, it emphasizes that different beams should be weighted according to the expected range of signal strengths, which is very useful in practice systems and is closely related to our current work. Since the signal strengths of GNSS signals are almost equal, we can define the weights corresponding to the desired GNSS signals are the same when compared with this work.

In this paper, we propose a novel STPAP beamforming algorithm based on MVDR criterion for GNSS receivers, which can achieve a balance between the output C/N_0_ performance and computational complexity. The contributions of our work are as follows: (a) A novel constraint vector for the STPAP architecture, which only requires a single adaptive filter or few adaptive filters when multiple GNSS satellites exist in the FOV, is proposed. For simplicity, since the proposed STPAP algorithm based on the MVDR criterion which constrains multiple satellites at once, we can call it MVDR-CMS criterion in this paper. Consequently, it can reduce the computational complexity effectively compared with the traditional STPAP algorithm based on the criteria in [30]; (b) The number of the desired GNSS signals processed in a single adaptive filter is discussed, so it will not cause drastic output C/N_0_ performance degradation when obtaining a lower computational complexity. Especially, the output C/N_0_ performance of the proposed STPAP algorithm based on the MVDR-CMS criterion is close to that of the existing STPAP algorithm based on the MVDR-CSS criterion in [30] if the parameter J, which denotes the number of GNSS satellites that processed by the same adaptive filter, is proper. (c) An adaptive algorithm based on the CLMS method is derived to calculate the weight vector for the proposed STPAP beamforming algorithm. 

The paper is organized as follows. Section 2 presents the polarization concept and the STPAP architecture. In Section 3, a novel STPAP beamforming algorithm based on the MVDR criterion is proposed. Besides, an adaptive algorithm to calculate the weight vector is derived for the proposed STPAP beamforming algorithm. In Section 4, simulations are carried out to validate the effectiveness of the proposed algorithm. Moreover, the proposed STPAP beamforming algorithm is compared with the existing STPAP algorithms based on the criterions in [29,30,31]. Finally, Section 5 concludes this paper.

## 2. Mathematical Model

### 2.1. Polarization Mode

**Figure 1:**
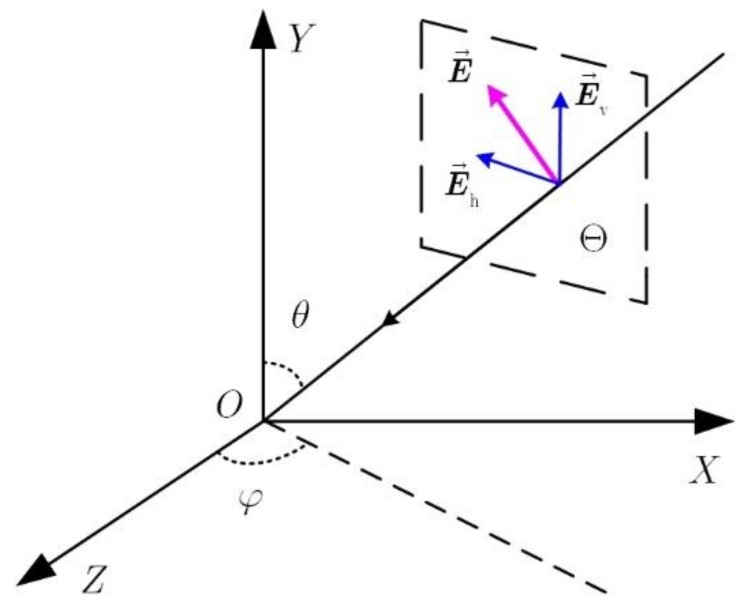
Transverse electric wave.

As depicted in Figure 1, a transverse electric (TE) wave is incident from the direction (θ,φ) with respective to the reference point O, where θ∈[0,π/2] represent the pitching angle and φ∈[0,2π) denote the azimuth angle. Moreover, TE wave refers to an electromagnetic wave in which the electric field is perpendicular to the propagation direction. The pitching angle refers to the acute angle between the direction of the incoming signal and the normal of the antenna. The azimuth angle refers to the angle between the projection of the incoming signal on the antenna and the reference direction, which is artificially specified. Define the transient electric field vector in plane Θ as $\vec{E}(t)$ and it can be written as

(1) $$\vec{E}(t)=\xi_{h}(t){\vec{E}}_{h}+\xi_{v}(t){\vec{E}}_{v},$$

where $\left({\vec{E}}_{h},{\vec{E}}_{v}\right)$ represents a pair of the orthonormal vector in the plane Θ, and $\xi_{h}(t)$, $\xi_{v}(t)$ respectively denote the transient projection values along the ${\vec{E}}_{h}$ and ${\vec{E}}_{v}$.

According to the orientation of the end point of the transient electric field vector, the TE wave can be classified into linear polarization, circular polarization, and elliptical polarization (EP). Moreover, linear polarization can be classified into horizontal polarization (HP) and vertical polarization (VP). Circular polarization can be classified into right-handed circular polarization (RHCP) and left-handed circular polarization (LHCP). To describe the polarization mode of the TE wave, the polarization parameter (γ,η) is adopted where γ∈[0,π/2] represents the amplitude ratio between the horizontal component and the vertical component of the electric field and meanwhile η∈[0,2π) denotes the phase difference between the horizontal component and the vertical component of the electric field. The polarization modes mentioned above can be defined by the polarized parameters in Table 1.

The polarization information of the TE wave can be distinguished by the electric vector sensor (EVS). Complete EVS contains three concentric and mutually perpendicular dipoles, while the dual-polarized EVS that consists of a pair of crossed dipoles is utilized most widely in practice. Note that multiple dual-polarized EVSs arrayed in space can form the DPSA. The TE wave received by the dual-polarized EVS can be given by

(2) $$x(t)=\left[\begin{array}{c} s_{x}(t)\\ s_{z}(t) \end{array}\right]=\left[\begin{array}{cc} -\sin\varphi & \cos\theta \cos\varphi \\ \cos\varphi & \cos\theta \sin\varphi \end{array}\right]\left[\begin{array}{c} \cos\gamma \\ \sin\gamma e^{j\eta } \end{array}\right]s(t),$$

where $s_{x}(t)$ is the signal received by the dipole along the x axis, $s_{z}(t)$ is the signal received by the dipole along the z axis, and s(t) denotes the envelope of the incident TE wave. One can find that the received information is not only determined by the polarization of the TE wave, but also related to the DOA of the incident signal.

### 2.2. STPAP Architechture

**Figure 2:**
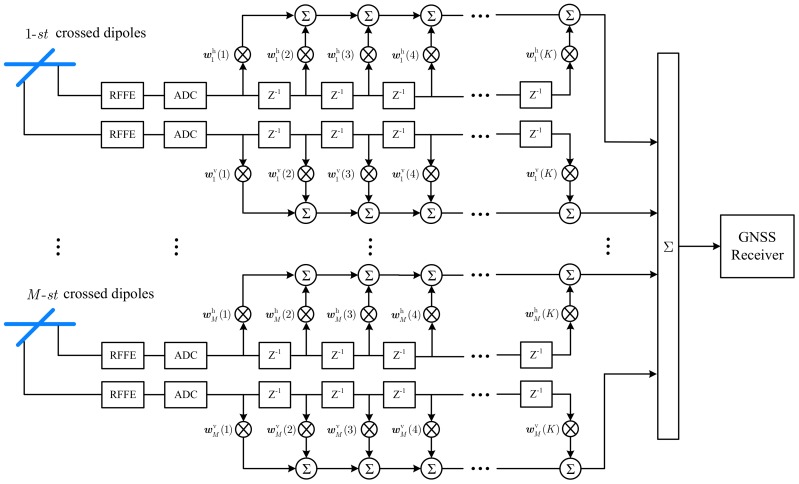
The STPAP architecture using the DPSA.

Consider a DPSA consisting of M pairs of crossed dipoles. A block diagram of the STPAP architecture is shown in Figure 2. The signal received by each dipole is firstly down converted to intermediate frequency in the radio frequency front end (RFFE) and then digitized by an analog to digital converter (ADC). Assume that each dipole is followed by a tapped delay line (TDL) with K taps and a delay of $T_{0}$ seconds between taps, with which a block of K time domain samples are acquired from each dipole. After that, the received signal is processed by the proposed STPAP beamforming algorithm and then sent into the GNSS receiver for acquisition.

Let $X(n)\in {\mathbb{C}}^{2MK\times 1}$ denotes the received signal for the n-th block

(3) $$X(n)=[X_{1}(n);X_{2}(n);\cdots ;X_{K}(n)]$$

with

(4) $$X_{k}(n)={\left[x_{1}^{h}(t-(k-1)T_{0}),x_{1}^{v}(t-(k-1)T_{0}),\cdots ,x_{M}^{h}(t-(k-1)T_{0}),x_{M}^{h}(t-(k-1)T_{0})\right]}^{H},$$

where ℂ represents the set of the complex numbers, H denotes the conjugate transpose, and $x_{m}^{h}(t)$, $x_{m}^{v}(t)$ are the most recent time domain samples. In addition, $x_{m}^{h}(t-(k-1)T_{0})$ and $x_{m}^{v}(t-(k-1)T_{0})$ (m=1,2,⋯,M,k=1,2,⋯K) denote the time domain sample of the m-th horizontal dipole and the m-th vertical dipole at the k-th tap, respectively.

Define the weight vector $W(n)\in {\mathbb{C}}^{2MK\times 1}$ for the n-th block as

(5) $$W(n)=[W_{1}(n);W_{2}(n);\cdots ;W_{M}(n)]$$

with

(6) $$W_{k}(n)={\left[w_{1}^{h}(k),w_{1}^{v}(k),\cdots ,w_{M}^{h}(k),w_{M}^{v}(k)\right]}^{H},$$

where $w_{m}^{h}(k)$ denotes the weight value for the m-th horizontal dipole at the k-th tap and $w_{m}^{v}(k)$ denotes the weight value for the m-th vertical dipole at the k-th tap.

With (3) and (5), the output for the n-th block is presented as

(7) $$\begin{array}{ll} Y(n) & =W^{H}(n)X(n)\\ & =\sum_{m=1}^{M}\sum_{k=1}^{K}[w_{m}^{h}(k)x_{m}^{h}(t-(k-1)T_{0})+w_{m}^{v}(k)x_{m}^{v}(t-(k-1)T_{0})]. \end{array}$$

## 3. Proposed STPAP Beamforming Algorithm

### 3.1. Novel MVDR-Based Criterion

Assume that L desired GNSS signals are incident from angular direction $\left(\theta_{l},\varphi_{l}\right)$ with polarized parameter $\left(\gamma_{l},\eta_{l})(l=1,2,\cdots ,L\right)$ and Q interferences are incident from angular direction $\left(\theta_{q},\varphi_{q}\right)$ with polarized parameter $\left(\gamma_{q},\eta_{q})(q=1,2,\cdots ,Q\right)$. Using the DPSA for the STPAP, the received signal model for the n-th block can be denoted as

(8) $$X(n)=\sum_{l=1}^{L}A_{l}(\theta_{l},\varphi_{l},\gamma_{l},\eta_{l})s_{l}(n)+\sum_{q=1}^{Q}A_{q}(\theta_{q},\varphi_{q},\gamma_{q},\eta_{q})s_{q}(n)+G(n),$$

where $s_{l}(n)={\left[s_{l}(t),\cdots ,s_{l}(t-(K-1)T_{0})\right]}^{H}$ and $s_{q}(n)={\left[s_{q}(t),\cdots ,s_{q}(t-(K-1)T_{0})\right]}^{H}$ are respectively the complex envelope of the l-th desired GNSS signal and the q-th interference for the n-th block, $A_{l}(\theta_{l},\varphi_{l},\gamma_{l},\eta_{l})$ and $A_{q}(\theta_{q},\varphi_{q},\gamma_{q},\eta_{q})$ respectively denote the joint space–time-polarization steering vector of the l-th desired GNSS signal and q-th interference, and $G(n)\in {\mathbb{C}}^{2MK\times 1}$ represents the additive white Gaussian noise vector for the n-th block.

Due to the aforementioned STPAP architecture, the joint space–time-polarization steering vector $A(\theta ,\varphi ,\gamma ,\eta )\in {\mathbb{C}}^{2MK\times 1}$ is given by

(9) $$A(\theta ,\varphi ,\gamma ,\eta )=I_{K\times K}⊗a_{s}(\theta ,\varphi )⊗a_{p}(\theta ,\varphi ,\gamma ,\eta )$$

with

(10) $$\begin{array}{l} a_{s}(\theta ,\varphi )={\left[\begin{array}{ccccc} 1 & e^{-j\Delta \phi (\theta ,\varphi )} & e^{-j2\Delta \phi (\theta ,\varphi )} & \cdots & e^{-j(M-1)\Delta \phi (\theta ,\varphi )} \end{array}\right]}^{H},\\ a_{p}(\theta ,\varphi ,\gamma ,\eta )=\left[\begin{array}{c} -\sin\varphi \cos\gamma +\cos\theta \cos\varphi \sin\gamma e^{j\eta }\\ \cos\varphi \cos\gamma +\cos\theta \sin\varphi \sin\gamma e^{j\eta } \end{array}\right], \end{array}$$

where ⊗ denotes the Kronecker product and $I_{K\times K}$ is the K-th order identity matrix. In addition, $a_{s}(\theta ,\varphi )$ and $a_{p}(\theta ,\varphi ,\gamma ,\eta )$ respectively represent the spatial steering vector and polarized steering vector, Δϕ(θ,φ) denotes the phase difference between the adjacent elements, and $j=\sqrt{-1}$. 

The traditional MVDR criterion is designed to minimize the array output power subject to the constraint that keeping the array gain towards the desired GNSS satellites undistorted and its expression is given by

(11) $$\begin{array}{l} \underset{W(n)}{\mathrm{Minimize}}\;W^{H}(n)R(n)W(n)\\ s.t.\;\;\;\;\;\;\;\;W^{H}(n)C=1\;\;\;\;n=1,2,\cdots N, \end{array}$$

where $C\in {\mathbb{C}}^{2MK\times 1}$ represents the constraint vector and $R(n)\in {\mathbb{C}}^{2MK\times 2MK}$ is the covariance matrix. In practice, it is infeasible to acquire the theoretical covariance matrix and thus it can be replaced with the sample covariance matrix $\tilde{R}(n)=\frac{1}{N}\sum_{n=1}^{N}X(n)X^{H}(n)$, in which N is the number of blocks.

In this paper, a novel constraint vector $\bar{C}\in {\mathbb{C}}^{2MK\times J}$ for the STPAP, which keeps the array gain towards J desired GNSS satellites in the FOV undistorted at once while minimizing the array output power, is proposed. We can give the expression of $\bar{C}$ as

(12) $$\bar{C}=\left[\begin{array}{cccc} c_{h,1,1}^{} & c_{h,1,2} & \cdots & c_{h,1,J}\\ c_{v,1,1} & c_{v,1,2} & \cdots & c_{v,1,J}\\ \vdots & \vdots & \vdots & \vdots \\ c_{h,M,1} & c_{h,M,2} & \cdots & c_{h,M,J}\\ c_{v,M,1} & c_{v,M,2} & \cdots & c_{v,M,J}\\ 0 & 0 & \cdots & 0\\ \vdots & \vdots & \vdots & \vdots \\ 0 & 0 & \cdots & 0 \end{array}\right]$$

with

(13) $$\begin{array}{ll} c_{h,m,l} & =a_{s,m}(\theta_{l},\varphi_{l})a_{p,h}(\theta_{l},\varphi_{l},\gamma =\pi /4,\eta =\pi /2)\\ & =(-\sin\varphi_{l}\cos\frac{\pi }{4}+\cos\theta_{l}\cos\varphi_{l}\sin\frac{\pi }{4}e^{j\frac{\pi }{2}})e^{-j(m-1)\Delta \phi (\theta_{l},\varphi_{l})}, \end{array}$$

(14) $$\begin{array}{ll} c_{v,m,l} & =a_{s,m}(\theta_{l},\varphi_{l})a_{p,v}(\theta_{l},\varphi_{l},\gamma =\pi /4,\eta =\pi /2)\\ & =(\cos\varphi_{l}\cos\frac{\pi }{4}+\cos\theta_{l}\sin\varphi_{l}\sin\frac{\pi }{4}e^{j\frac{\pi }{2}})e^{-j(m-1)\Delta \phi (\theta_{l},\varphi_{l})}, \end{array}$$

where $a_{s,m}(\theta_{l},\varphi_{l})$ denotes the m-th component of the spatial steering vector corresponding to the l-th GNSS signal. In addition, $a_{p,h}(\theta_{l},\varphi_{l},\gamma =\pi /4,\eta =\pi /2)$ and $a_{p,v}(\theta_{l},\varphi_{l},\gamma =\pi /4,\eta =\pi /2)$ represents the horizontal and vertical component of the polarized steering vector corresponding to the l-th GNSS signal, respectively. Note that the DOAs of the desired GNSS signals, $\left(\theta_{l},\varphi_{l}\right)$, are assumed to be known as a priori through the existing GNSS DOA estimation methods proposed in [32,33,34,35], such as the inertial navigation device assisting method. Moreover, the polarized parameters for all desired GNSS signals are (γ,η)=(π/4,π/2) as the polarization modes of all GNSS signals are RHCP. 

Furthermore, the parameter J is worth discussing because it determines the number of adaptive filters that used in the proposed STPAP beamforming algorithm when multiple GNSS satellites exist in the FOV. When J=L, only one adaptive filter is required for all the desired GNSS satellites in the FOV; When 2≤J≤L−1, at least two but no more than L−1 adaptive filters is needed to deal with all the desired GNSS satellites in the FOV; When J=1, the proposed MVDR-CMS criterion degenerates into the MVDR-CSS criterion in [22]. Therefore, the parameter J is defined as 2≤J≤L in the proposed MVDR-CMS criterion. Obviously, the computational complexity of the proposed criterion is lower than that of the MVDR-CSS criterion in [22].

With the novel constraint vector in (12), the expression in (11) can be rewritten as

(15) $$\begin{array}{l} \underset{W_{\mathrm{all}}(n)}{\mathrm{Minimize}}\;W_{\mathrm{all}}^{H}(n)\tilde{R}(n)W_{\mathrm{all}}(n)\\ s.t.\;\;\;\;\;\;\;\;W_{\mathrm{all}}^{H}(n)\bar{C}={\vec{1}}_{J}\;\;\;\;n=1,2,\cdots N, \end{array}$$

where $W_{\mathrm{all}}(n)\in {\mathbb{C}}^{2MK\times 1}$ denotes the weight vector for the J GNSS satellites in the FOV and ${\vec{1}}_{J}$ a 1×J vector containing all ones. Moreover, the weight vector $W_{\mathrm{all}}(n)$ are found from the solution to (14) and it can be presented as $W_{\mathrm{all}}(n)={\tilde{R}}^{-1}(n)\bar{C}[{\bar{C}}^{H}{\tilde{R}}^{-1}(n)\bar{C}]{\vec{1}}_{J}^{H}.$ It is noticed that the weight vector $W_{\mathrm{all}}(n)$ can be applied to the J desired GNSS satellites in the FOV at once, which can greatly reduce the computational complexity, especially as the parameter J increases. 

For comparison, the aforementioned MVDR-CSS criterion in [22] for the STPAP when using the DPSA is presented as

(16) $$\begin{array}{l} \underset{W_{l}(n)}{\mathrm{Minimize}}\;W_{l}^{H}(n)\tilde{R}(n)W_{l}(n)\\ s.t.\;\;\;\;\;\;\;\;W_{l}^{H}(n){\overset{⌢}{C}}_{l}=1\;\;\;\;n=1,2,\cdots N \end{array}$$

with

(17) $${\overset{⌢}{C}}_{l}={\left[\begin{array}{cccccccc} c_{h,1,l} & c_{v,1,l} & \cdots & c_{h,M,l} & c_{v,M,l} & 0 & \cdots & 0 \end{array}\right]}^{H},$$

where $W_{l}(n)\in {\mathbb{C}}^{2MK\times 1}$ represents the weight vector for the l-th GNSS signal and its expression can be written as $W_{l}(n)={\tilde{R}}^{-1}(n){\overset{⌢}{C}}_{l}[{\overset{⌢}{C}}_{l}^{H}{\tilde{R}}^{-1}(n){\overset{⌢}{C}}_{l}].$ Besides, ${\overset{⌢}{C}}_{l}\in {\mathbb{C}}^{2MK\times 1}$ denotes the constraint vector for the l-th GNSS signal, which indicates that L adaptive filters are required if there are L GNSS satellites in the FOV and thus it will definitely increase the burden of computation. Thus, it is easily found that the performance of the proposed MVDR-CMS criterion is better than that of the MVDR-CSS criterion in terms of the computational complexity. It is noticed that the theoretical analysis is based on the joint space–time-polarization architecture, while it can also be applied to the space architecture and space–time architecture when the polarization discriminator is removed.

### 3.2. Adaptive Algorithm for Calculating the Weight Vector

The direct inverse matrix (DMI) method or constraint least mean square (CLMS) method can be usually adopted as adaptive algorithms to implement the proposed MVDR-CMS criterion for the STPAP. The DMI method is sample in form but it is very computationally intensive due to the inversion of matrix. In comparison, the CLMS method has a lower computational complexity, which is feasible to calculate the weight vector $W_{\mathrm{all}}$ iteratively in this paper. Therefore, the iterative expression to acquire the weight vector for the proposed STPAP beamforming algorithm will be described in the following work. 

With (15), the iterative function based on the steepest descent method can be presented as

(18) $$W_{\mathrm{all}}(i+1)=W_{\mathrm{all}}(i)-\mu \nabla_{W_{\mathrm{all}}}F(W_{\mathrm{all}}),$$

where i represents the iteration number, μ denotes a fixed step factor, and $\nabla_{W_{\mathrm{all}}}F(W_{\mathrm{all}})$ represents the gradient function. Moreover, function $F(W_{\mathrm{all}})$ is the combination of the cost function and constraint function in (15) and it can be given by

(19) $$F=\frac{1}{2}W_{\mathrm{all}}^{H}\tilde{R}W_{\mathrm{all}}+B_{\lambda }{\left(W_{\mathrm{all}}^{H}\bar{C}-{\vec{1}}_{J}\right)}^{H},$$

where $B_{\lambda }$ is the Lagrange multiplier and the coefficient 1/2 is adjoined to simplify calculation. Note that the Lagrange multiplier $B_{\lambda }$ for the proposed MVDR-CMS criterion is not a constant but a 1×J vector, which varies with iteration.

Taking the gradient of (19) with respective to $W_{\mathrm{all}}$, the gradient function $\nabla_{W_{\mathrm{all}}}F(W_{\mathrm{all}})$ can be presented as

(20) $$\nabla_{W_{\mathrm{all}}}F(W_{\mathrm{all}})=\tilde{R}W_{\mathrm{all}}+\bar{C}B_{\lambda }^{H}.$$

Substituting (19) into (18), the iterative function can be rewritten as

(21) $$W_{\mathrm{all}}(i+1)=W_{\mathrm{all}}(i)-\mu \tilde{R}(i)W_{\mathrm{all}}(i)-\mu \bar{C}B_{\lambda }^{H}(i),$$

where $W_{\mathrm{all}}(i+1)$ must satisfy the constraint function in (15) and thus

(22) $$W_{\mathrm{all}}^{H}(i+1)\bar{C}={\vec{1}}_{J}.$$

Then, substituting (21) into (22), we can obtain the Lagrange multiplier $B_{\lambda }(i)$ as

(23) $$B_{\lambda }(i)=\frac{1}{\mu }[W_{\mathrm{all}}^{H}(i)\bar{C}{\left({\bar{C}}^{H}\bar{C}\right)}^{-1}-\mu W_{\mathrm{all}}^{H}(i)\tilde{R}(i)\bar{C}{\left({\bar{C}}^{H}\bar{C}\right)}^{-1}-{\vec{1}}_{J}^{H}{\left({\bar{C}}^{H}\bar{C}\right)}^{-1}].$$

Using (23), (21) can be expressed as

(24) $$\begin{array}{ll} W_{\mathrm{all}}(i+1) & =W_{\mathrm{all}}(i)-µ\tilde{R}(i)W_{\mathrm{all}}(i)-\bar{C}[W_{\mathrm{all}}^{H}(i)\bar{C}{\left({\bar{C}}^{H}\bar{C}\right)}^{-1}-\\ & µW_{\mathrm{all}}^{H}(i)\tilde{R}(i)\bar{C}{\left({\bar{C}}^{H}\bar{C}\right)}^{-1}-{\vec{1}}_{J}^{H}{\left({\bar{C}}^{H}\bar{C}\right)}^{-1}]{}^{H}\\ & =W_{\mathrm{all}}(i)-µ\tilde{R}(i)W_{\mathrm{all}}(i)-\bar{C}{\left({\bar{C}}^{H}\bar{C}\right)}^{-1}{\bar{C}}^{H}W_{\mathrm{all}}(i)+\\ & µ\bar{C}{\left({\bar{C}}^{H}\bar{C}\right)}^{-1}{\bar{C}}^{H}\tilde{R}(i)W_{\mathrm{all}}(i)+\bar{C}{\left({\bar{C}}^{H}\bar{C}\right)}^{-1}{\vec{1}}_{J}^{H}\\ & =T[W_{\mathrm{all}}(i)-µ\tilde{R}(i)W_{\mathrm{all}}(i)]+V \end{array}$$

with

(25) $$\begin{array}{l} T=I-\bar{C}{\left({\bar{C}}^{H}\bar{C}\right)}^{-1}{\bar{C}}^{H},\\ V=\bar{C}{\left({\bar{C}}^{H}\bar{C}\right)}^{-1}{\vec{1}}_{J}^{H} \end{array}$$

where $I\in {\mathbb{C}}^{2MK\times 2MK}$ denotes the identity matrix. Note that the matrix T and V do not change with iteration. 

Furthermore, the error signal can be given by

(26) e(i)=d(i)−y(i)

with

(27) $$y(i)=W_{\mathrm{all}}^{H}(i)X(i),$$

where e(i) represents the i-th iterative error, y(i) denotes the *i*-th iterative output data, $X(i)\in {\mathbb{C}}^{2MK\times 1}$ represents the i-th input time domain sample block, and d(i) is the reference signal. d(i) denotes the reference signal, which is the one corresponding to the signals after the first ADC in Figure 2. 

**Figure 3:**
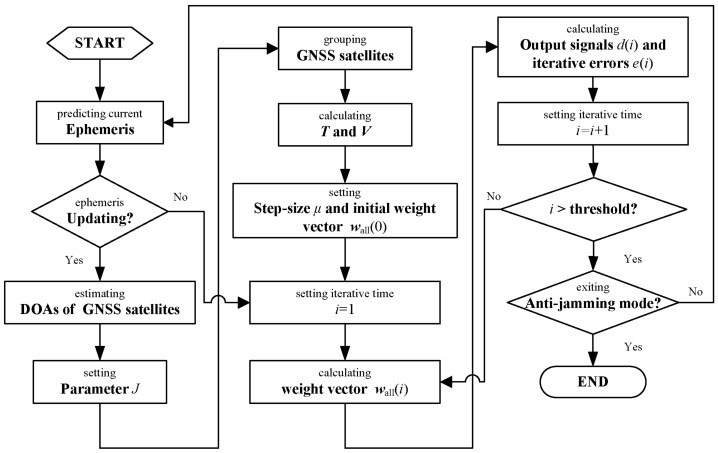
Flow chart of the proposed algorithm.

According to the above description, the flow chart of the proposed algorithm can be depicted as Figure 3. Note that it is necessary to regularly check whether ephemeris is updated. When the ephemeris is updated, the GNSS satellites in the FOV should be regrouped and meanwhile ***T*** and ***V*** should also be recalculated.

## 4. Simulation Results

In this section, the effectiveness of the proposed STPAP beamforming algorithm based on the MVDR-CMS criterion is validated firstly. Then, the output C/N_0_ and computational complexity performance of the proposed STPAP algorithm based on the MVDR-CMS criterion are compared with those of the PM criterion in [29] and the MVDR-CSS criterion in [30], respectively. To observe the array pattern in the whole frequency band clearly, the linear uniform DPSA is used preferentially. Thus, an eight-element linear uniform DPSA with half wavelength spacing is utilized and each dipole of the DPSA is followed by a TDL with eight taps. The desired GNSS signal is BeiDou-2 (BD2) signal at B3 band whose carrier frequency is 1268.52 MHz and mainlobe bandwidth is 20.46 MHz. The analog intermediate frequency is 46.52 MHz. Moreover, the sampling frequency and the number of samples are set as 62 MHz and 62,000, respectively. The received digital signal is firstly generated and then a quadrature down-conversion mixer is adopted to acquire baseband I/Q signals. After that, a low-pass filter is used to remove the noise that is out of the mainlobe. Finally, the proposed STPAP beamforming algorithm can be applied to process the signal. 

Three scenarios have been designed and the simulation parameters are presented in Table 2. Note that 20 Monte-Carlo simulations, in which the locations of interferences vary from one to the next as shown in Table 2, are carried out to get averages of the results in scenarios 2 and 3.

(a) Scenario 1: Without loss of generality, two desired BD2 signals and one wideband interference with EP are involved in this scenario. To validate the effectiveness of the proposed STPAP algorithm, we can observe whether null forms towards the interference or beams form towards the two desired GNSS signals in the joint space–time-polarization domain. Moreover, the BD2 software receiver is also utilized to validate that the desired GNSS signals can be acquired.

(b) Scenario 2: Twelve desired BD2 signals and four wideband interferences are generated. The output C/N0 performance of the proposed STPAP algorithm is compared with those of the existing STPAP algorithms based on the criterions in [29,30,31].

(c) Scenario 3: To verify that the proposed algorithm can be also valid when the polarization discrimination is removed, all the twelve desired BD2 signals and four wideband interferences are assumed to be RHCP. Moreover, the output C/N_0_ performance of the proposed STPAP algorithm is also compared with those of the existing STPAP algorithms based on the criterions in [29,30,31].

### 4.1. Effectiveness Validation of the Proposed STPAP Beamforming Algorithm

#### 4.1.1. Array Pattern in the Joint Space–Time-Polarization Domain

In this scenario, the effectiveness of the proposed STPAP beamforming algorithm is validated through observing the array pattern in the joint space–time-polarization domain. It is noticed that the array pattern corresponding to the STPAP algorithm are related to the spatial, temporal and polarized parameters and thus it is a four-dimensional data in this simulation, which means that it is difficult to depict the data in only one figure. As a result, if nulls in one domain are supposed to be observed, we have to fix parameters in another two domains. Since the data of the space domain and time domain in the simulation is one-dimensional, the array pattern in the space domain and time domain can be observed together if the polarized parameter has been fixed. Meanwhile, array pattern in the polarization domain can be observed if the spatial and temporal parameters are fixed. In addition, we can choose 5.115 MHz as the fixed temporal parameter, which also applies to the next scenario, as all interferences and desired BD2 signals are wideband. Note that the normalized frequency, which refers to the ratio between the actual frequency and the maximum frequency, is adopted in this simulation for simplicity. Specifically, the maximum frequency is set as 20.46 MHz that is identical to the frequency band of the BD2 signal in the simulation.

**Figure 4:**
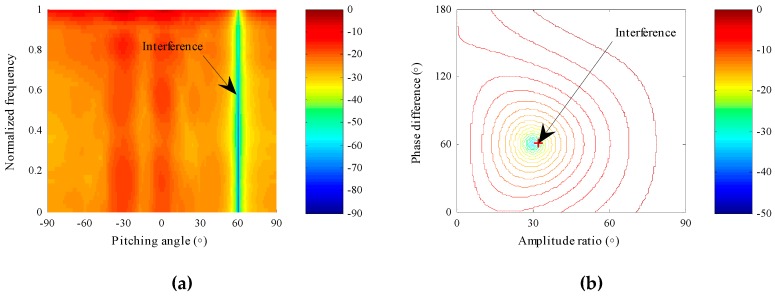
Array patterns when parameters are fixed as that of the interference using the proposed STPAP beamforming algorithm. (**a**) Null forms in the space domain and time domain towards the interference. (**b**) Null forms in the polarization domain towards the interference.

Using the proposed STPAP beamforming algorithm, as depicted in Figure 4a, a null forms at the incident direction of π/3 in the whole frequency band when the polarized parameter is set as that of the interference. Moreover, as shown in Figure 4b, when the spatial parameter and temporal parameters are fixed as those of the interference, a null forms in the polarization domain at (π/6, π/3), which is exactly the polarized parameter of the interference. According to the results in Figure 4, one can obtain that the proposed STPAP beamforming algorithm can successfully form nulls towards the interference in the joint space–time-polarization domain.

**Figure 5:**
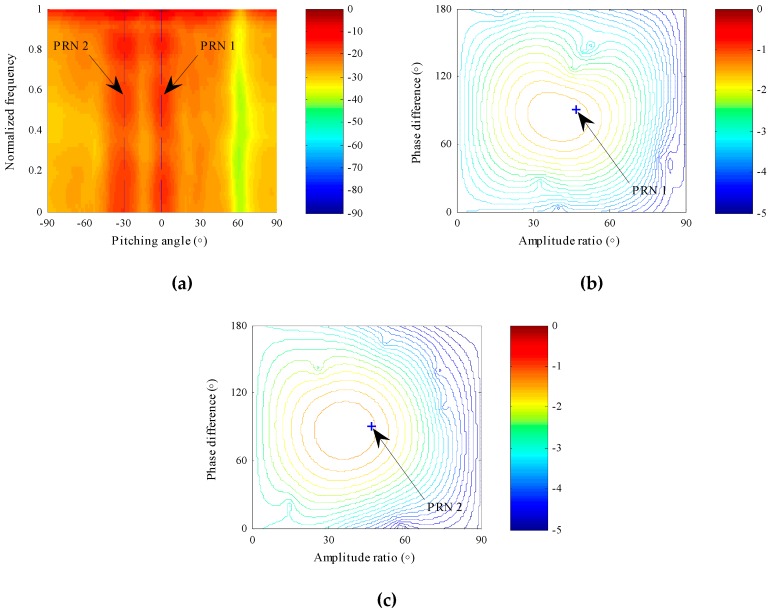
Array patterns when parameters are respectively fixed as those of the two desired BD2 signals using the proposed STPAP beamforming algorithm. (**a**) Beam forms in the space domain and time domain towards the two BD2 signals. (**b**) Beam forms in the polarization domain towards the BD2 signal of PRN 1. (**c**) Beam forms in the polarization domain towards the BD2 signal of PRN 2.

Furthermore, we can also observe whether beams form towards the two desired BD2 signals using the proposed STPAP beamforming algorithm. As depicted in Figure 5a, beams form in the space domain and time domain when the polarized parameter is set as that of the desired BD2 signal. Similarly, beams form in the polarization domain when the spatial parameter and temporal parameters are respectively fixed as those of the two desired BD2 signals, as shown in Figure 5b,c. The results obviously indicate that the desired BD2 signals are well preserved when the interference is cancelled in the joint space–time-polarization domain with the proposed STPAP beamforming algorithm. 

#### 4.1.2. Signal Acquisition in the BD2 Software Receiver 

**Figure 6:**
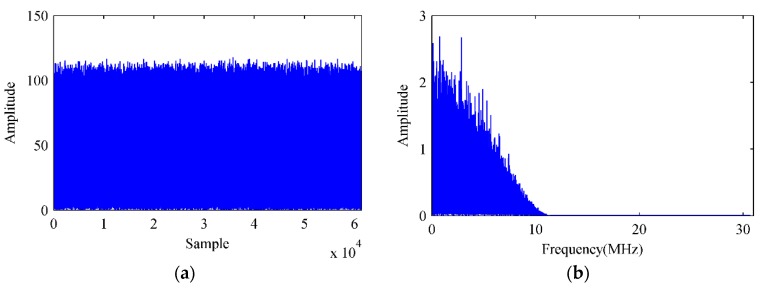
Time domain samples and single-sided amplitude spectrum of the input baseband signal. (**a**) Time domain samples. (**b**) Single-sided amplitude spectrum.

As mentioned above, the input signal received by the DPSA is firstly down converted into baseband, whose time domain samples and single-sided amplitude spectrum are shown in Figure 6. As the interferences are generated by the DSSS technique in the simulation, they have the same single-sided amplitude spectrum forms with the BD2 signal. 

**Figure 7:**
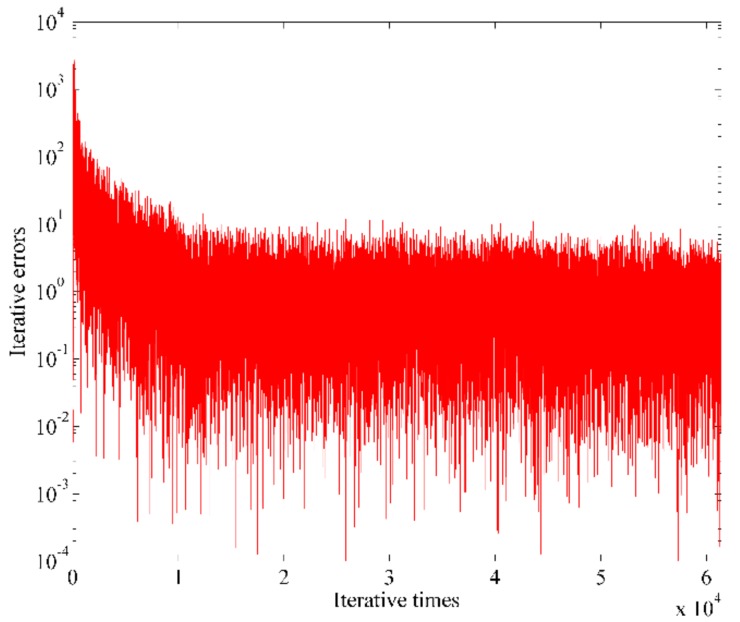
Iterative error signals changing with the iterative times.

**Figure 8:**
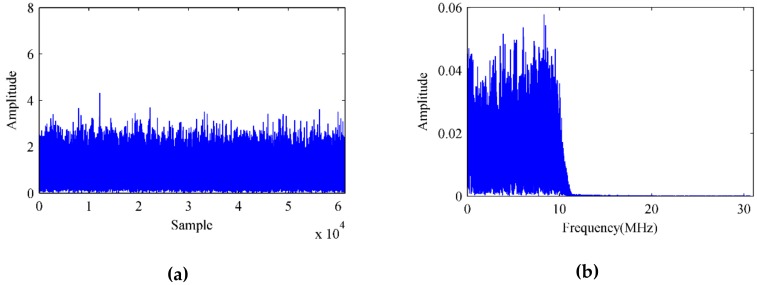
Time domain samples and single-sided amplitude spectrum of the output baseband signal using the proposed STPAP beamforming algorithm. (**a**) Time domain samples. (**b**) Single-sided amplitude spectrum.

Using the proposed adaptive algorithm in Section 3.2 for the proposed STPAP beamforming algorithm and meanwhile setting the step-size µ as 5 × 10^−7^, the iterative error signals are shown in Figure 7, from which it can be seen that the error values tend to stabilize as the number of iterations increase. After that, the time domain samples and single-sided amplitude spectrum corresponding to the output baseband signal can be depicted in Figure 8.

**Figure 9:**
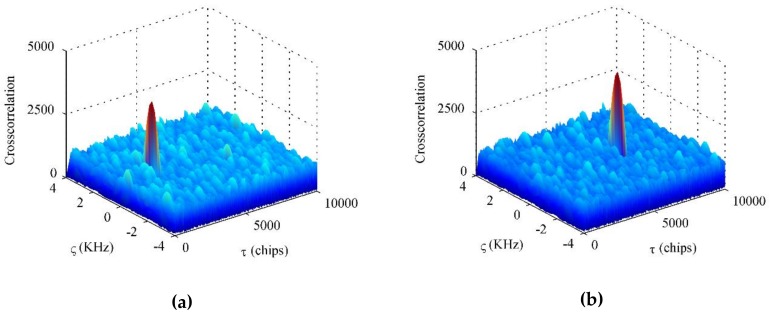
Acquisition results corresponding to the desired BD2 satellites using the proposed STPAP beamforming algorithm: (**a**) PRN 1; (**b**) PRN 2.

Furthermore, the output baseband signal is sent into the BD2 software receiver. Assume that ς denotes Doppler frequency and τ represents chips. The acquisition results are depicted in Figure 9, in which the two desired BD2 navigation satellites have been acquired successfully. 

According to the simulation results in Section 4.1, we can find that the proposed STPAP beamforming algorithm is effective in canceling interferences and forming beams towards the desired BD2 satellites in the joint space–time-polarization domain. Moreover, the received signal processed by the proposed algorithm can be acquired by the BD2 software receiver.

### 4.2. Output C/N_0_ Performance when Interferences with Different Polarization Modes

**Figure 10:**
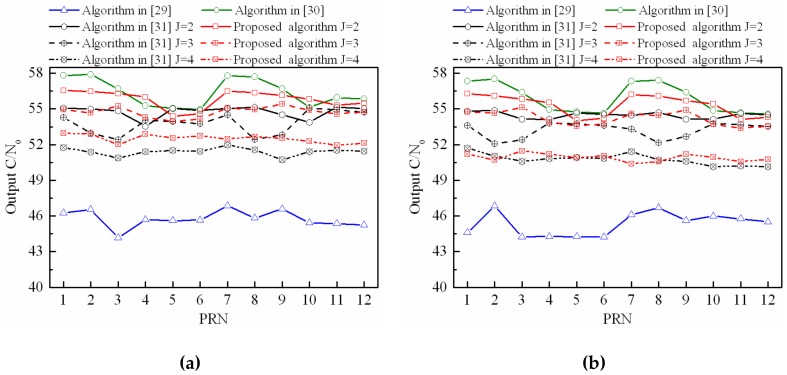

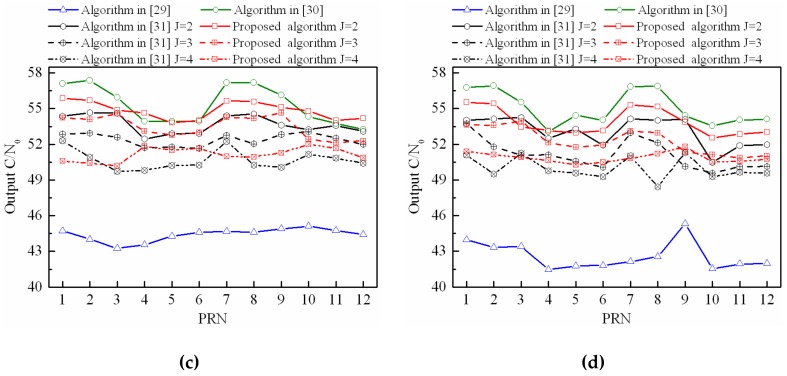
Output C/N_0_ performance comparison between the proposed STPAP algorithm and the STPAP algorithms based on the criterions in [29,30,31] when there are interferences with different polarization modes: (**a**) one interference; (**b**) two interferences; (**c**) three interferences; (**d**) four interferences.

In this scenario, the output C/N_0_ performance of the proposed STPAP algorithm is compared with those of the STPAP algorithms based on the criterions in [29,30,31] when there are multiple GNSS satellites in the FOV. Without loss of generality, we can compare the output C/N_0_ performance of these four algorithms when one beam covers two, three, and four BD2 satellites, respectively when there are respectively one, two, three, and four interferences. As shown in Figure 10, the results can be summarized as follows: (a) the output C/N_0_ performance of the STPAP algorithm in [30] is always the best one, while the output C/N_0_ performance of the STPAP algorithm in [29] is worse than the other three criteria; (c) the output C/N_0_ performance of the proposed STPAP algorithm is a little better than the existing STPAP algorithm in [31]. Meanwhile, it is close to that of the existing STPAP algorithm in [30] when one beam covers two or three satellites. It indicates that the parameter J in (12) is better to be set as 2 or 3 to prevent the output C/N_0_ performance from degrading dramatically.

### 4.3. Output C/N0 Performance When Interferences with the Same Polarization Mode

**Figure 11:**
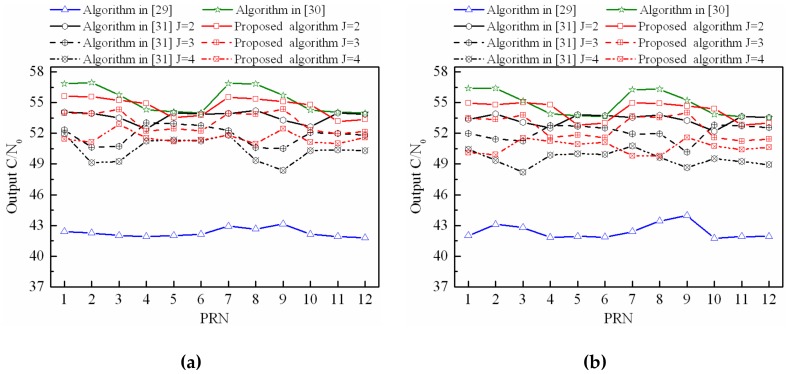

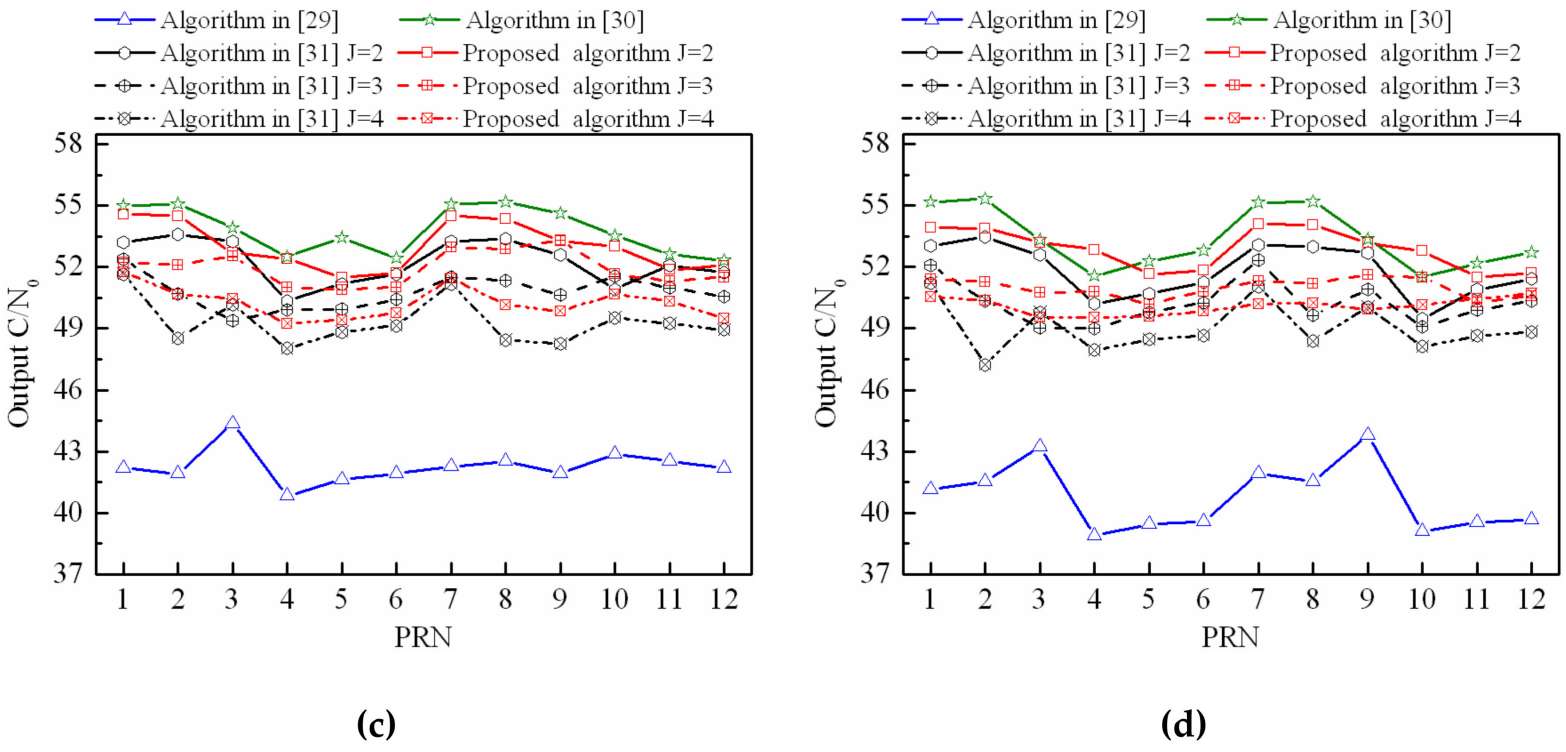
Output C/N_0_ performance comparison between the proposed STPAP algorithm and the STPAP algorithms based on the criterions in [29,30,31] when there are interferences with the same polarization mode: (**a**) one interference; (**b**) two interferences; (**c**) three interferences; (**d**) four interferences.

In Section 4.2, a lot of space has been allocated to polarization discrimination. To emphasize the performance of the proposed algorithm, all the 12 satellite signals and four wideband interferences are assumed to have the same polarization mode, which is RHCP. Analogously, the output C/N_0_ performance of the proposed STPAP algorithm based on the MVDR-CMS criterion is compared with those of the STPAP algorithms based on the criterions in [29,30,31] when there are multiple GNSS satellites in the FOV. As depicted in Figure 11, the results are identical to those in Section 4.2. 

### 4.4. Computational Complexity Performance

In this simulation, we will focus on the computational complexity performance of the proposed STPAP algorithm and the existing STPAP algorithm based on the criterions in [30,31] since they have the similar processing flow. As for the existing STPAP algorithm based on the PM criterion in [29], its processing flow is different form the MVDR-based criterion because it is unnecessary to estimate DOAs of the desired GNSS signals, due to which it has the lowest computational complexity and thus it is meaningless considering this algorithm in this section.

The number of addition and multiplication can be adopted to measure the computational complexity. Using the proposed adaptive iterative algorithm described in Section 3.2 to implement the proposed STPAP algorithm and the existing STPAP algorithm based on the criterions in [30,31], the number of addition and multiplication that consumed in each iteration can be shown in Table 3. It is noticed that J GNSS satellites can be processed at once with only one adaptive filter using the proposed STPAP algorithm and the existing STPAP algorithm based on the criterion in [31], while J adaptive filters are required using the existing STPAP algorithm in [30]. Thus, it should be taken into consideration when calculating the computational complexity. Moreover, when the desired GNSS satellites change, the metrics T and V in (24) are required to be recalculated. Since the update frequency of the desired GNSS satellites is not so fast with regard to the iterative convergence progress, we can assume that the desired GNSS satellites will not change during an iterative period as shown in Figure 3, due to which the calculation amount of T and V can be ignored for simplicity. Moreover, one can obtain that the computational complexity of the proposed STPAP algorithm is almost equal to that of the existing STPAP algorithm based on the criterion in [31] with the assumption that the calculation amount of T and V can be ignored.

**Figure 12:**
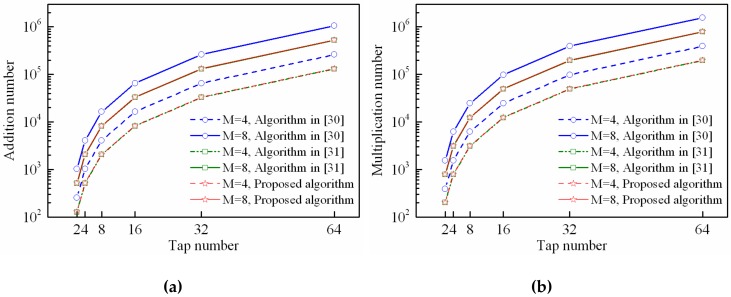
Computational complexity of the proposed STPAP beamforming algorithm and the STPAP algorithms based on the criterions in [30,31] when J=2: (**a**) addition number; (**b**) multiplication number.

**Figure 13:**
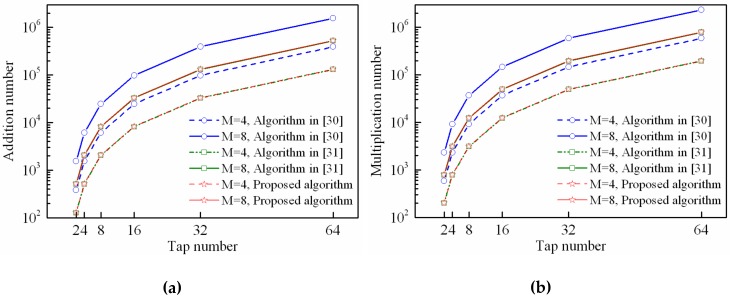
Computational complexity of the proposed STPAP beamforming algorithm and the STPAP algorithms based on the criterions in [30,31] when J=3: (**a**) addition number; (**b**) multiplication number.

As depicted in Figure 12 and Figure 13, it can be seen that the number of addition and multiplication that used in the proposed STPAP algorithm and the existing STPAP algorithm in [31] are always less than that of the existing STPAP algorithm based on the criterion in [30] when there are different tap number and element number. The results indicate that the proposed STPAP beamforming algorithm has a lower computational complexity than the traditional STPAP algorithm based on the MVDR-CSS criterion in [30]. 

From the results in the simulation, we can make a conclusion that the proposed STPAP beamforming algorithm based on the MVDR-CMS criterion can effectively reduce the computational complexity and meanwhile keep the output C/N_0_ performance close to that of the traditional STPAP algorithm based on the MVDR-CSS criterion in [30] when the parameter J is fixed as 2 or 3. Moreover, although the computational complexity of the existing STPAP algorithm in [31] is almost equal to that of the proposed STPAP beamforming algorithm, its output C/N_0_ performance is not better than that of the proposed STPAP beamforming algorithm.

## 5. Conclusions

A novel STPAP beamforming algorithm based on the MVDR-CMS criterion, which can achieve a balance between the output C/N_0_ performance and computational complexity, has been proposed in this paper. With the assumption that the DOAs of the desired GNSS satellites are known a priori through existing DOA estimation methods, the proposed STPAP algorithm based on the MVDR-CMS criterion is designed to process multiple GNSS satellites with a single adaptive filter, which is beneficial to reduce computational complexity compared with the traditional STPAP algorithm based on the MVDR-CSS criterion in [30]. Meanwhile, the output C/N_0_ performance of the proposed STPAP algorithm based on the MVDR-CMS criterion is close to that of the MVDR-CSS criterion when the parameter J that denotes the number of GNSS satellites processed in one adaptive filter is proper. Concretely speaking, J is better to be set as two or three, which means that an adaptive filter preferably processes two or three GNSS satellites at once. Besides, an adaptive algorithm based on the CLMS method is derived to calculate weight vector iteratively for the proposed STPAP beamforming algorithm, which can avoid the calculation of matrix inversion. In addition, the proposed STPAP algorithm can be still effective when the polarization discriminator is removed. 

## Figures and Tables

**Table 1:** Polarized parameter.

Polarization Mode	Amplitude Ratio (γ)	Phase Difference (η)
HP	0	0,π
VP	π/2	0,π
RHCP	π/4	π/2
LHCP	π/4	−π/2
EP	[0,π/2]	[−π,π)

**Table 2:** Simulation parameters

Scenario	Incident Signal	SNR/INR	Spatial Parameter	Polarized Parameter	
				Amplitude Ratio	Phase Difference
1	BD2 B3 (PRN 1)	−20 dB	0	π/4	π/2
	BD2 B3 (PRN 2)	−20 dB	−π/6	π/4	π/2
	Wideband interference (EP)	40 dB	π/3	π/6	π/3
2	BD2 B3 (PRN 1)	−20 dB	π/36	π/4	π/2
	BD2 B3 (PRN 2)	−20 dB	π/12	π/4	π/2
	BD2 B3 (PRN 3)	−20 dB	π/4	π/4	π/2
	BD2 B3 (PRN 4)	−20 dB	5π/12	π/4	π/2
	BD2 B3 (PRN 5)	−20 dB	4π/9	π/4	π/2
	BD2 B3 (PRN 6)	−20 dB	17π/36	π/4	π/2
	BD2 B3 (PRN 7)	−20 dB	−π/36	π/4	π/2
	BD2 B3 (PRN 8)	−20 dB	−π/12	π/4	π/2
	BD2 B3 (PRN 9)	−20 dB	−π/4	π/4	π/2
	BD2 B3 (PRN 10)	−20 dB	−5π/12	π/4	π/2
	BD2 B3 (PRN 11)	−20 dB	−4π/9	π/4	π/2
	BD2 B3 (PRN 12)	−20 dB	−17π/36	π/4	π/2
	Wideband interference (HP)	40 dB	[−5π/36, −7π/36]	0	π
	Wideband interference (VP)	40 dB	[11π/36, 13π/36]	π/2	π
	Wideband interference (LHCP)	40 dB	[5π/36, 7π/36]	π/4	−π/2
	Wideband interference (RHCP)	40 dB	[−11π/36, −13π/36]	π/4	π/2
3	BD2 B3 (PRN 1)	−20 dB	π/36	π/4	π/2
	BD2 B3 (PRN 2)	−20 dB	π/12	π/4	π/2
	BD2 B3 (PRN 3)	−20 dB	π/4	π/4	π/2
	BD2 B3 (PRN 4)	−20 dB	5π/12	π/4	π/2
	BD2 B3 (PRN 5)	−20 dB	4π/9	π/4	π/2
	BD2 B3 (PRN 6)	−20 dB	17π/36	π/4	π/2
	BD2 B3 (PRN 7)	−20 dB	−π/36	π/4	π/2
	BD2 B3 (PRN 8)	−20 dB	−π/12	π/4	π/2
	BD2 B3 (PRN 9)	−20 dB	−π/4	π/4	π/2
	BD2 B3 (PRN 10)	−20 dB	−5π/12	π/4	π/2
	BD2 B3 (PRN 11)	−20 dB	−4π/9	π/4	π/2
	BD2 B3 (PRN 12)	−20 dB	−17π/36	π/4	π/2
	Wideband interference (RHCP)	40 dB	[−5π/36, −7π/36]	π/4	π/2
	Wideband interference (RHCP)	40 dB	[11π/36, 13π/36]	π/4	π/2
	Wideband interference (RHCP)	40 dB	[5π/36, 7π/36]	π/4	π/2
	Wideband interference (RHCP)	40 dB	[−11π/36, −13π/36]	π/4	π/2

**Table 3:** Computational complexity.

Criterion	Addition Number	Multiplication Number
Proposed STPAP algorithm	$8M^{2}K^{2}$	$12M^{2}K^{2}+2MK$
The existing algorithm in [30]	$8M^{2}K^{2}J$	$12M^{2}K^{2}J+2MKJ$
The existing algorithm in [31]	$8M^{2}K^{2}$	$12M^{2}K^{2}+2MK$
